# Editorial: Recent Advances and Future Perspectives for Agavoideae Research: *Agave, Yucca* and Related Taxa

**DOI:** 10.3389/fpls.2021.687596

**Published:** 2021-05-10

**Authors:** Luis E. Eguiarte, James Leebens-Mack, Karolina Heyduk

**Affiliations:** ^1^Laboratorio de Evolución Molecular y Experimental, Departamento de Ecología Evolutiva, Instituto de Ecología, Universidad Nacional Autónoma de México, Mexico City, Mexico; ^2^Department of Plant Biology, University of Georgia, Athens, GA, United States; ^3^School of Life Sciences, University of Hawai'i at Mãnoa, Honolulu, HI, United States

**Keywords:** arid and semiarid climates, biofuel, desert, hybridization, mescal, physiological adaptations, pollination mutualism, pulque

The Agavoideae (Asparagaceae) are a group of charismatic plants native to the Americas (if including the genus *Hosta*, also Asia) that are very diverse in their ecology and comprise 12 genera and ca. 445 species. Species in the Agavoideae display impressive adaptions in order to live in arid and semiarid conditions, have been historically very important for local human populations, and today are gaining increased attention due to their immense biotechnological potential (Davis et al., 2011; Cushman et al., 2015). In this collection we include 15 papers concerning a variety of cutting-edge studies on different aspects of the biology of the Agavoideae, which together illuminate their evolution and uniqueness.

Papers in this collection focus on the two largest genera in the Agavoidae: *Yucca* and *Agave*. Hybridization is not uncommon in *Yucca*, despite a long history of specialized pollination mutualism in this genus (Pellmyr and Leebens-Mack, 1999; McKain et al., 2016). Royer et al. described a hybrid zone in the *Y. brevifolia* and *Y. jaegeriana* (Joshua trees) from the Mojave desert that are pollinated by different *Tegeticula* moth species. The *Yucca* species present different sizes, with *Y. brevifolia* taller and with larger flowers than *Y. jaegeriana*, and morphological differences exist between their pollinators as well. Royer et al. analyzed a narrow (ca. 4 km in width) hybrid zone between these species in the Tikaboo Valley, southern Nevada, using RAD-seq data, microsatellite loci, chloroplast variation, vegetative and floral traits, and pollinator frequency. They found overlapping genomic and pollinator clines, consistent with a narrow hybrid zone generated by strong selection, but with wider phenotypic and a chloroplast clines.

In the Baja California peninsula, Arteaga et al. studied the populations of two closely related *Yucca* species: *Y. valida* that lives mostly in the central section of the peninsula and *Y. capensis* that is only found in the southern tip of the peninsula. They confirmed the hybrid origin of geographically and morphologically intermediate populations using nextRAD derived SNP along Principal Components and Structure analysis and Approximate Bayesian computation simulations. This conclusion was supported by the distribution models constructed using climatic data for the present and the past.

In the Atlantic coast of North America, Heyduk et al. studied the hybrid origin *of Y. gloriosa*, a putatively hybrid species between *Y. aloifolia* and *Y. filamentosa*, which is photosynthetically intermediate (Rentsch and Leebens-Mack, 2012; Heyduk et al., 2016). The authors used whole genome shotgun data to assemble complete chloroplast genomes in the three species. They found that *Y. gloriosa* chloroplast haplotypes were nested in three separate clades: one related to *Y. filamentosa*, and two related to *Y. aloifolia*, supporting a hybrid but complex origin for *Y. gloriosa*. They also analyzed the transposons in their nuclear sequencing reads: while overall repetitive content varied between the three species, expression patterns showed little increased transcriptional activity of transposons in *Y. gloriosa*, suggesting that no transposon release occurred in the hybrid.

Jolly et al. described different morphological and physiological adaptations to deal with the dry conditions in *Y. brevifolia* and *Hesperoyucca whipplei*, two species from the Mojave desert. The authors suggested that the ability of *H. whipplei* to adjust both its vein density and stomatal density allows for higher gas exchange, thus permitting this species to grow in drier conditions than *Y. brevifolia*.

Most of the papers in this collection concentrate in studies of different species in *Agave*. Jiménez-Barron et al. analyzed the phylogeny, divergence times, and speciation rates in *Agave sensu lato*, a clade containing more than 250 species. They concluded that the genus is organized in different main clades. One clade is formed by the Striatae group, which is the sister group to the rest of the *Agave sensu lato*. Another clade is formed by the herbaceous taxa *Manfreda, Polianthes*, and *Prochnyanthes*, that diverged from other linages within *Agave sensu lato* ca. 3.55 Ma. Within *Agave*, they found two significant diversification shifts: one soon after the origin of *Agave sensu lato*, at ca. 6.18 Ma, and a second within *Agave sensu stricto*, ca. 2.68 Ma.

The *Agave* genus is not only fascinating because of its diversity and adaptions, but also because these plants were central for the development of the American native cultures and civilizations. Ortiz-Cano et al. reviewed their physiological adaptations to dry environments and their traditional uses, in particular for making drinks like mescal, tequila, and pulque, and their potential new applications as alternative crops for biofuel production. The authors focused on the Hohokam, a Pre-Columbian Indigenous People from the Sonoran desert that used “rock mulching” (i.e., rock piles) that helped to cultivate agaves by harvesting during rainfall and retaining soil moisture.

Cabrera-Toledo et al. analyzed the levels of morphological and genetic variation and differentiation patterns in *A. maximiliana*, used in the production of raicilla, a local kind of mescal in Western-Central Mexico. The authors found strong morphological and genetic differentiation between populations (*F*_ST_ = 0.43), and isolation by distance in the genetic markers. The authors compared plants in different management categories (monoculture, managed forestry systems, and wild populations), but they did not find differences in morphology nor in genetic diversity among these categories. Torres-García et al. studied the demography of *A. inaequidens*, another species used in the production of raicilla and other types of mescal, across four wild populations in Central Western Mexico. The study indicated that analyzed wild populations are stable, concluding that extraction rates from 10 to 30% of mature individuals for making mescal could be sustainable, but only if 200 to 300 agave plants of the younger sizes are introduced every year into the populations.

Another group of agaves used for the production of pulque–a traditional Mexican alcoholic beverage produced through open fermentation of agave sap—was studied by Trejo et al. They analyzed vegetative traits of landraces of *A. salmiana* and *A. mapisaga* in Tlaxcala, a highland state in central east Mexico, and their genetic relationships using a chloroplast region (*trnL*) and nuclear ITS sequences. Both, data on the morphology and genetic markers of the landraces aligned with the species classification, with the exception of *A. salmiana* subsp. *salmiana* “Ayoteco,” which is more related to *A. mapisaga* var. *mapisaga*. They concluded that low intensity artificial selection together with gene flow and plasticity could explain the high number of phenotypically similar landraces.

*Agave kerchovei* has a restricted distribution, mainly in the Tehuacán -Cuicatlán Valley, in the states of Oaxaca and Puebla. Aguirre-Planter et al. studied the chloroplast variation and both current and past ecological niche models, finding high levels of total chloroplast genetic variation and very strong genetic differentiation (i.e., *F*_ST_ = 0.92). They suggest that the Pleistocene glacial cycles played a critical role in changing the distribution and population sizes of the species.

The interaction with other species seems to be very important in the evolutionary ecology of agavoid species, in particular mutualistic pollinator interactions (e.g., the *Tegeticula* moth in *Yucca* and the nectar feeding *Leptonycteris* bats in *Agave;* Pellmyr and Leebens-Mack, 1999; McKain et al., 2016: Eguiarte et al., 2021). But microbes, including both fungi and bacteria, also seem to play very important roles in the biology of the Agavoideae. In *A. lechuguilla*, in Cuatro Ciénegas, in the Chihuahuan desert, López-Lozano et al. analyzed the microbial communities in the soil and in its rhizosphere using 16S rRNA gene sequences. Actinobacteria were more abundant in the soil, while Proteobacteria dominated in the rhizosphere. The authors reported differences in bacterial diversity, community composition, potential functions of the bacteria taxa, and interaction networks between the soil and *A. lechuguilla* rhizosphere.

The plants of the Agavoideae have many other physiological and biochemical adaptations and unique chemical profiles. Lledías et al. isolated, characterized and analyzed the molecular function and evolution of mayahuelin, a protein that is abundant in the central spike of the rosette in *A. tequilana* var. *azul* and in other agaves, making up to 20% or even more of the total protein. The protein is a type I Ribosome inactivating protein (RIP). The authors also used this gene to identify accessions closely related to *A. tequilana* var. *azul*. Agaves store their carbohydrates in the form of fructan polymers, instead of starch or sucrose. Pérez-López and Simpson describe the fructans in *Agave* that provide a source of carbohydrates for the transition from vegetative to reproductive stages; for this reason, inflorescences are removed to avoid depletion of fructan reserves in cultivated and managed populations of *Agave*.

The striking physiological adaptations of *Agave* for living in dry areas are highlighted in the study of Jones et al. where they conducted field experiments in *A. americana* to evaluate their ethanol yield potential, analyzing their efficiency in water use, their carbohydrates production, and the products obtained by enzymatic hydrolysis comparing with other plants—grasses–used to produce biofuels. They concluded that *A. americana* was able to produce important amounts of soluble carbohydrates by using very little water, and thus have an important potential for future biofuel production.

Finally, this collection also includes a methodological paper by González-Gutiérrez et al. that describes how to analyze the F-actinin in plant species with thick tissues, as is the case for Agavoideae plants.

This collection analyses only a few species, adaptations and uses from a very large group of fascinating plants, including agaves called “el árbol de las maravillas” (the tree of wonders) by José de Acosta (de Acosta, 1608), a Jesuits missionary and, among yuccas, Joshua Tree described as “The most repulsive tree in the Vegetable Kingdom” by American explorer Lt. John C. Fremont in 1844 (we disagree). We hope that this collection of studies will help to increase the awareness of the Agavoideae, and inspire future work on the group's striking diversity of form and ecological function.

## Author Contributions

LE, JL, and KH proposed the Research Topic, edited manuscripts, and wrote the editorial. All authors contributed to the article and approved the submitted version.

## Conflict of Interest

The authors declare that the research was conducted in the absence of any commercial or financial relationships that could be construed as a potential conflict of interest. The reviewer CM declared a shared affiliation, with no collaboration, with one of the authors, LE, to the handling editor at the time of review.
